# Association of rs6983267 at 8q24, HULC rs7763881 polymorphisms and serum lncRNAs CCAT2 and HULC with colorectal cancer in Egyptian patients

**DOI:** 10.1038/s41598-017-16500-4

**Published:** 2017-11-24

**Authors:** Olfat G. Shaker, Mahmoud A. Senousy, Eman M. Elbaz

**Affiliations:** 1 Medical Biochemistry and Molecular Biology Department, Faculty of Medicine, Cairo University, Cairo, Egypt; 2 Biochemistry Department, Faculty of Pharmacy, Cairo University, Cairo, Egypt

## Abstract

The impact of HULC rs7763881 on colorectal cancer (CRC) susceptibility is not yet known. Also, the biological function of the cancer-related rs6983267 remains unclear. We investigated the association of these SNPs with the risk of CRC and adenomatous polyps (AP), their correlation with CCAT2 and HULC expression, and the potential of serum CCAT2 and HULC as biomarkers for CRC. 120 CRC patients, 30 AP patients, and 96 healthy controls were included. Genotyping and serum lncRNAs were assayed by qPCR. Studied SNPs were not associated with AP susceptibility. rs6983267 GG was associated with increased CRC risk, whereas rs7763881 AC was protective. rs7763881 and rs6983267 CT haplotype was protective. Serum CCAT2 and HULC were upregulated in CRC and AP patients versus controls and discriminated these groups by ROC analysis. rs6983267 GG and rs7763881 AA patients demonstrated higher serum CCAT2 and HULC compared with GT/TT and AC, respectively. rs6983267 and serum HULC predicted CRC diagnosis among non-CRC groups (AP + controls) by multivariate analysis. Studied SNPs or serum long noncoding RNAs weren’t correlated with nodal or distant metastasis. In conclusion, rs6983267 and rs7763881 are potential genetic markers of CRC predisposition and correlate with serum CCAT2 and HULC, two novel potential non-invasive diagnostic biomarkers for CRC.

## Introduction

Colorectal cancer (CRC) is one of the most commonly diagnosed cancers worldwide^1^. In Egypt, CRC incidence is keeping highly in recent years, and interestingly in young age^2^. Patients with advanced CRC have poor prognosis, but early detection improves clinical outcome^3^. Therefore, improving the early diagnosis and treatment of CRC is urgently needed.

The genesis of CRC involves complex multi-factorial steps in which an interplay exists between environmental factors, genetic background, represented by single nucleotide polymorphisms (SNPs), and abnormal gene expression^4^, but knowledge of the full molecular basis of CRC is still limited^5^. Elucidating the molecular mechanisms underlying CRC development and progression may unravel new diagnostic and prognostic biomarkers and therapeutic targets for CRC.

Several cancer risk loci are transcribed into long noncoding RNAs (lncRNAs, >200 nucleotides)^6^. These lncRNAs regulate various epigenetic, transcriptional, and post-transcriptional events, and potentially contribute to carcinogenesis as tumor suppressors or oncogenes^5,6^. Indeed, several lncRNAs are aberrantly expressed in CRC^5^. In addition, genome wide association studies have identified genetic variants in lncRNAs genomic regions as candidate risk factors for CRC^7–9^. These SNPs were postulated to alter lncRNA expression and/or structure, or affect lncRNA pathways.

Colon cancer-associated transcript 2 (CCAT2) is a 340 nt lncRNA transcribed from the 8q24 genomic region that encompass the SNP rs6983267^10^. CCAT2 is upregulated in CRC tissues particularly in cases of metastatic cancer^11^, and is highly overexpressed in microsatellite-stable CRC tumors, where it enhances chromosomal instability, tumor invasion and metastasis by enhancing WNT signaling and upregulating MYC and MYC-activated miRNAs (miR-17-5p and miR-20)^10^. The SNP rs6983267, located in a gene desert in the MYC enhancer region, has been identified as being associated with increased CRC risk^7,8,10^, and was functionally linked to enhanced WNT signaling in CRC^12^. In addition, a correlation of rs6983267 genotypes with MYC as well as CCAT2 expression in CRC tissues has been reported, but with controversy^10,11,13,14^. However, the association of rs6983267 with tumor aggressiveness and its correlation with CCAT2 level are unclear. Furthermore, the  role of rs6983267 in predisposing CRC risk in the Egyptian population is not yet investigated.

Highly upregulated liver cancer (HULC) is a 500 nt lncRNA that regulates cell invasion and metastasis by acting as a miR-372 sponge^15^. HULC is upregulated in CRC tumors, and was associated with CRC progression and metastasis by silencing the expression of the tumor suppressor NKD2^16^. The SNP rs7763881, mapped to the HULC gene located at the 6p24.3 region, was previously reported as a decreased risk factor for hepatocellular carcinoma^17^ and esophageal cancer^18^. However, the impact of rs7763881 on CRC susceptibility, its involvement in regulating HULC expression, and its relationship with tumor-related data are not yet known.

Therefore, we aimed to investigate the association of rs6983267 at 8q24 and HULC rs7763881 SNPs with the susceptibility of CRC as well as adenomatous polyps (AP), the commonest premalignant lesions for CRC, their relationship with clinicopathological data, and their correlation with serum CCAT2 and HULC expression. As the clinical relevance of serum CCAT2 and HULC as biomarkers of CRC was not previously studied, we also investigated serum CCAT2 and HULC expression in AP and CRC patients, their correlation with clinicopathological data, and their potential as non-invasive biomarkers of CRC.

## Results

### Demographic and clinicopathological features of the studied groups

Demographic, laboratory, colonoscopic‚ and pathological features of the studied groups are shown in Table 1. All diagnosed cases of CRC and AP were sporadic. Patients with AP were significantly younger than those with CRC (*P* < 0.05). Gender was not significantly different (*P* = 0.67); however, there was a male predominance in CRC and AP patients representing 66.7% and 60%, respectively. 40% and 28.5% of CRC and AP patients were smokers, respectively.

**Table 1:** Demographic and clinicopathological data of the studied groups.

	CRC	AP	Healthy controls	*P* value
	(n = 120)	(n = 30)	(n = 96)	
Age (years)	50.95 ± 11.93 ^A^	42.12 ± 17.26^B^	47.98 ± 9.85^AB^	**0.001**
Sex, n (%)				0.67
Male	80 (66.7)	18 (60)	57 (59.4)	
Female	40 (33.3)	12 (40)	39 (40.6)	
Hemoglobin (g/dl)	10.72 ± 2.84 ^A^	12.24 ± 1.38^B^		**0.005**
Platelet count × 10^3^/mm^3^	277.8 ± 94.48 ^A^	248.8 ± 40 ^A^		0.1
(150–400 × 10^3^/mm^3^)				
TLC × 10^3^/mm^3^	7.29 ± 2.46 ^A^	6.08 ± 1.03^B^		**0.009**
(4–11 × 10^3^/mm^3^)				
ESR (mm/hr)	47 ± 34.48 ^A^	20 ± 15^B^		<**0.0001**
CEA (ng/ml)	44.18 ± 56.77 ^A^	2.75 ± 1.4^B^		**0.0001**
**Colonoscopy**				
Polyps	16 (13.3)	30 (100)		
Fungating mass	82 (68.3)	0 (0)		
Hyperemia	3 (2.5)	0 (0)		
Annular stricture	32 (26.7)	0 (0)		
Malignant ulcer	6 (5)	0 (0)		
**Anatomical site**				
Proximal colon	48 (40)	6 (20)		
Distal colon	40 (33.3)	11 (36.7)		
Rectum	32 (26.7)	11 (36.7)		
Whole colon	0 (0)	2 (6.6)		
**Tumor grade**				
Adenocarcinoma	116 (96.67)			
Well-differentiated	2 (1.72)			
Moderately differentiated	88 (75.86)			
Poorly-differentiated	26 (22.42)			
Adenosquamous carcinoma	2 (1.67)			
Undifferentiated carcinoma	2 (1.67)			
**Mucinous tumors**				
Yes	10 (9.6)			
No	106 (91.4)			
**Node metastasis**				
Present	44 (36.7)			
Absent	76 (63.3)			
**Distant metastasis**				
Present	20 (16.7)			
Absent	100 (83.3)			
**AJCC stage**				
Stage 0	0 (0)			
Stage I	18 (15)			
Stage II	58 (48.3)			
Stage III	24 (20)			
Stage IV	20 (16.7)			

Values are expressed as mean ± SD or number (percentage). AP, adenomatous polyps, CRC, colorectal cancer, CEA, carcinoembryonic antigen; ESR, erythrocyte sedimentation rate; TLC, total leukocyte count. *P* values in bold are statistically significant (*P* < 0.05). Groups with different letters are statistically significant.

Regarding colonoscopy, 68% of CRC patients were presented by fungating ulcerating masses, while the remaining were annular strictures and malignant rectal ulcers (26.7% and 5%, respectively). 73.3% of the CRC tumors were at the colon, the remaining were rectal, with the right colon was the most affected (40%).

Regarding histopathology, all CRC tumors were of variable sizes (≥5 cm). 96.6% of CRC tumors were adenocarcinoma, of which 22.42% were poorly differentiated and 9.6% were mucinous. Metastatic CRC was only observed in 16.7% of the studied patients; all were having hepatic focal lesions. 68.3% of CRC patients were diagnosed with AJCC stages II and III. In the AP group, 40% of patients had multiple (≥3) variable sized polyps, with the remaining with either one or 2 polyps and none of them belonged to any of the polyposis syndromes. Two-thirds of polyps were tubulovillous adenomas with only half of them showed dysplasia.

### Association of rs7763881 (A/C) and rs6983267 (G/T) with the risk of CRC and AP

Genotyping was processed without knowledge of the subjects’ case-control status. The concordance rates of repeated analyses using the same assay were 100% for the 2 SNPs. Minor allele frequencies (MAFs) in controls were similar to Ensembl GRCh37 release 89, 2017 for the 2 SNPs (Table S1). The distribution of the rs6983267 genotypes in the CRC group did not significantly deviate from Hardy-Weinberg equilibrium (*P* = 0.86) (Table S2).

The genotype and allele frequencies for rs7763881 and rs6983267 are shown in Table 2. For rs7763881, the genotype and allele frequencies were not significantly different between the AP patients and controls (*P* > 0.05). In CRC patients, the frequency of rs7763881 AC genotype was significantly lower than in healthy controls (73.3% vs 87.5%, respectively, crude *P* = 0.011) and associated with decreased CRC risk (AC vs AA, adjusted OR = 0.335, 95% CI = 0.157–0.716, *P* = 0.005) with adjustment for age and sex. However, there was no significant difference in the A and C allele frequencies between CRC patients and healthy controls (C vs A, OR = 0.744, 95% CI = 0.505–1.097, *P* = 0.14).

**Table 2:** Genotype and allele frequencies of rs7763881 (A/C) and rs6983267 (G/T) polymorphisms in CRC and adenomatous polyps compared to healthy controls.

Genotype and allele	Controls	CRC	Crude OR	*P*	Adjusted OR^a^	*P* ^a^	Polyps	Crude OR	*P*	Adjusted OR^a^	*P*
	n = 96 n (%)	n = 120 n (%)	(95% CI)		(95% CI)		n = 30 n (%)	(95% CI)		(95% CI)	
**HULC rs7763881 A/C**											
AA	12 (12.5)	32 (26.7)	r		r		6 (20)	r			
AC	84 (87.5)	88 (73.3)	0.393	**0.011**	0.335	**0.005**	24 (80)	0.57	0.37	0.512	0.24
			(0.189–0.813)		(0.157–0.716)			(0.19–1.68)		(0.166–1.58)	
CC	0 (0)	0	—	—	—	—	0 (0)	—	—		
A	108 (0.56)	152 (0.63)	r		—		36 (0.6)	r		—	
C	84 (0.44)	88 (0.37)	0.744	0.14	—	—	24 (0.4)	0.85	0.65	—	—
			(0.505–1.097)					(0.47–1.5)			
**rs6983267 G/T**											
GG	24 (25)	46 (38.3)	r		r		4 (13.3)	r		r	
GT	62 (64.6)	56 (46.7)	0.47	**0.016**	0.39	**0.005**	24 (80)	2.323	0.2	2.86	0.09
			(0.256–0.869)		(0.203–0.749)			(0.729–7.4)		(0.845–9.681)	
TT	10 (10.4)	18 (15)	0.939	1	0.863	0.766	2 (6.7)	1.2	1	1.1	0.924
			(0.375–2.35)		(0.327–2.275)			(0.188–7.64)		(0.149–8.1)	
TT vs GT vs GG				**0.03**					0.28		
GT/TT	72 (75)	74 (61.7)	0.536	**0.04**	0.47	**0.017**	26 (86.7)	2.167	0.22	2.698	0.1
			(0.297–0.968)		(0.254–0.872)			(0.686–6.84)		(0.807–9.024)	
GG/GT	86 (89.6)	102 (85)	r		r		28 (93.3)	r		r	
TT	10 (10.4)	18 (15)	1.5	0.41	1.63	0.26	2 (6.7)	0.61	1	0.564	0.48
			(0.665–3.462)		(0.698–3.804)			(0.126–2.97)		(0.113–2.8)	
G	110 (0.57)	148 (0.62)	r		—		32 (0.53)	r		—	
T	82 (0.43)	92 (0.38)	0.845	0.43	—	—	28 (0.47)	1.174	0.65	—	—
			(0.574–1.245)					(0.655–2.1)			

Values are expressed as number (percentage). CI, confidence interval; r, reference. *P* ^a^: adjusted for age and sex in a logistic regression model*. P* values in bold are statistically significant (*P* < 0.05).

For rs6983267, there were no significant differences in the genotype and allele frequencies between AP patients and controls (*P* > 0.05). In CRC, the genotype frequencies were significantly different between CRC and controls (*P* = 0.03), however, the allele frequencies were not significantly different between the two groups (T vs G, OR = 0.845, 95% CI = 0.574–1.245, *P* = 0.43). In the additive model, the GT genotype was protective against CRC (GT vs GG, adjusted OR = 0.39, 95% CI = 0.203–0.749, *P* = 0.005), while the minor homozygote TT genotype did not significantly affect the CRC risk (TT vs GG, adjusted OR = 0.863, 95% CI = 0.327–2.275, *P* = 0.766). In the dominant model, the common homozygote GG genotype was significantly associated with the risk of CRC (GG vs GT/TT, adjusted OR = 2.13, 95% CI = 1.146–3.937, *P* = 0.017). In the recessive model, the TT genotype was not a significant risk factor for CRC (TT vs GG/GT, adjusted OR = 1.5, 95% CI = 0.665–3.462, *P* = 0.41).

### Results of stratification analysis

In a stratification risk analysis by age and sex, the effects of rs7763881 A/C and rs6983267 G/T genotypes on CRC risk were further investigated (Table 3). rs7763881 AC was a protective candidate for CRC risk among male patients and younger patients (≤50 years) (AC vs AA, OR = 0.31, 95% CI = 0.117–0.825, *P* = 0.018; OR = 0.259, 95% CI = 0.094–0.716, *P* = 0.011, respectively). rs6983267 GG genotype was associated with increased CRC susceptibility among male patients and older patients (>50 years) (GG vs GT/TT, OR = 3.57, 95% CI = 1.53–8.33, *P* = 0.002; OR = 3.44, 95% CI = 1.25–10, *P* = 0.02, respectively). Conversely, the GT and GT/TT variants were associated with decreased CRC risk in these groups.

**Table 3:** Stratified analysis of the effect of rs7763881 (A/C) and rs6983267 (G/T) SNPs on CRC risk by age and sex.

Parameter	HULC rs7763881		*P*, OR (95% CI)	rs6983267				*P*, OR (95% CI)		
	AA	AC	(AC against AA)	GG	GT	TT	GT/TT	GT against GG	TT against GG	GT/TT against GG
	Cases/controls			Cases/controls						
**Age, n**										
>50	14/6	46/30	*P* = 0.605, 0.657 (0.227–1.9)	24/6	30/30	6/1	36/31	***P*** ** = 0.006**, 0.25 (0.089–0.698)	*P* = 1, 1.5 (0.15–14.94)	***P*** ** = 0.02**, 0.29 (0.1–0.8)
≤50	18/6	42/54	***P*** ** = 0.011**, 0.259 (0.094–0.716)	22/18	26/32	12/9	38/41	*P* = 0.41, 0.66 (0.297–1.494)	*P* = 1, 1.09 (0.375–3.167)	*P* = 0.56, 0.758 (0.353–1.627)
**Sex, n**										
male	22/6	58/51	***P*** ** = 0.018**, 0.31 (0.117–0.825)	32/9	36/38	12/10	48/48	***P*** ** = 0.003**, 0.26 (0.11–0.635)	*P* = 0.08, 0.337 (0.11–1.03)	***P*** ** = 0.002**, 0.28 (0.12–0.65)
female	10/6	30/33	*P* =0.4, 0.54 (0.176-1.683)	14/15	20/24	6/0	26/24	*P* = 1, 0.89 (0.34-2.28)	*P* = **0.027**	*P* = 0.81, 1.16 (0.46-2.9)

Values are expressed as numbers (cases/controls). *P* values in bold are statistically significant (*P* < 0.05).

### Joint effect and results of haplotype analysis

We examined the joint effect of studied gene polymorphisms in patients with CRC compared to control group (Table 4). Results revealed that the combined heterozygosity for rs7763881 and rs6983267 (AC + GT vs AA + GG, OR = 0.217, 95% CI = 0.057–0.817, *P* = 0.024) was a decreased risk factor for CRC. In addition, rs7763881 and rs6983267 CT haplotype was protective against CRC (CT vs AG, OR = 0.583, 95% CI = 0.388–0.877, *P* = 0.012).

**Table 4:** Haplotype and joint analysis of rs7763881 (A/C) and rs6983267 (G/T) polymorphisms in CRC patients compared to healthy controls.

Combined alleles	CRC, n (%)	Controls, n (%)	*P*	OR (95% CI)
AG	182 (38)	125 (32.5)	r	
AT	122 (25)	91 (23.75)	0.652	0.92 (0.646–1.312)
CG	114 (24)	95 (24.75)	0.318	0.824 (0.578–1.175)
CT	62 (13)	73 (19)	**0.012**	0.583 (0.388–0.877)
Combined genotypes				
AA + GG	12 (10)	3 (3.13)	r	
AA + GT	10 (8.33)	9 (9.38)	0.15	0.27 (0.058–1.313)
AA + TT	10 (8.33)	0 (0)	0.25	
AC + GG	34 (28.34)	21 (21.88)	0.23	0.404 (0.1–1.6)
AC + GT	46 (38.33)	53 (55.2)	**0.024**	0.217 (0.057–0.817)
AC + TT	8 (6.67)	10 (10.4)	0.072	0.2 (0.042–0.96)

Values are expressed in numbers. CRC, n = 120, healthy controls, n = 96*. P* values in bold are statistically significant (*P* < 0.05).

### Serum levels of HULC and CCAT2 in CRC and AP

**Figure 1:**
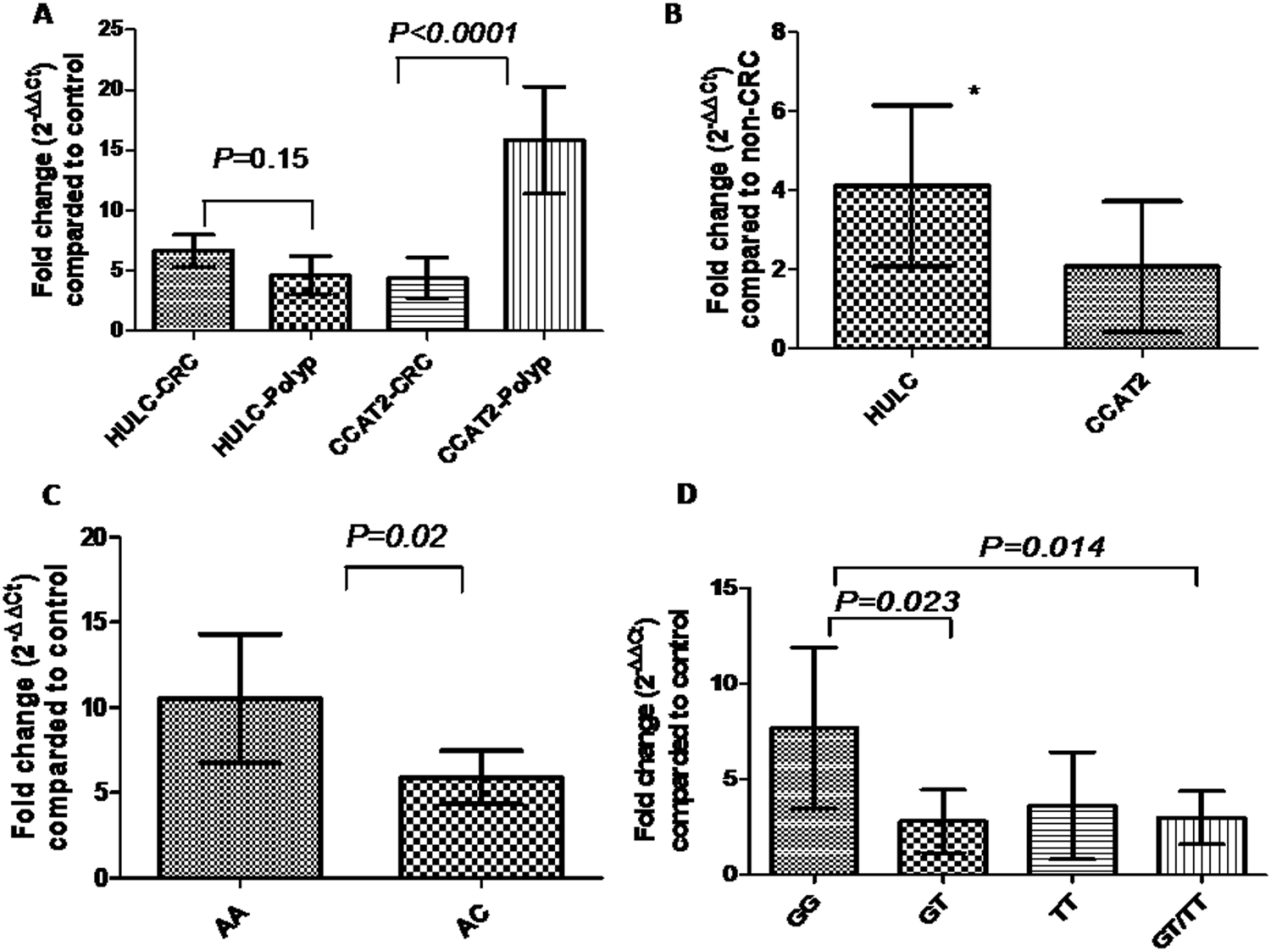
Serum expression levels of HULC and CCAT2. (**A**) Fold change of serum HULC and CCAT2 expression in patients with CRC (n = 120) and adenomatous polyps (n = 30) compared with healthy controls (n = 96), (**B**) Fold change of serum HULC and CCAT2 expression in CRC patients compared with non-CRC patients (healthy controls + adenomatous polyps patients, n = 126), (**C**) Expression of serum HULC in different HULC SNP rs7763881 genotypes (AA, n = 32, and AC, n = 88), and (**D**) Expression of serum CCAT2 in different rs6983267 genotypes (GG, n = 46, GT, n = 56, TT, n = 18, GT/TT, n = 74). Data are expressed as mean (95% CI). *means statistical significance (*P* < 0.05).

Serum HULC was significantly upregulated in CRC and AP patients compared with healthy controls with mean fold change of 6.76 (*P* < 0.0001) and 4.6 (*P* = 0.0002), respectively (Fig. 1A). Serum HULC levels were numerically higher in CRC than AP patients, but didn’t reach statistical significance (*P* = 0.15). Serum HULC was significantly higher in CRC compared to non-CRC groups (AP + healthy controls) with a mean fold change of 4.1 (*P* = 0.007) (Fig. 1B).

Serum CCAT2 was significantly upregulated in CRC and AP patients compared with healthy controls with mean fold change of 4.4 (*P* = 0.024) and 15.87 (*P* < 0.0001), respectively (Fig. 1A). Interestingly, serum CCAT2 levels were significantly higher in patients with AP than CRC patients (*P* < 0.0001), while serum CCAT2 was not significantly different between CRC and non-CRC groups (mean fold change 1.97, *P* = 0.1) (Fig. 1B).

### Effect of rs7763881 and rs6983267 genotypes on serum HULC and CCAT2 expression

We found that serum HULC level was significantly higher in CRC patients with rs7763881 AA than those with AC genotype (*P* = 0.02) (Fig. 1C). CRC patients with rs6983267 GG genotype were found to have higher expression level of serum CCAT2 than those with GT genotype (*P* = 0.023) or GT/TT genotypes (*P* = 0.014), however, serum CCAT2 levels were not significantly different between either patients with GG and TT or patients with GT and TT genotypes (*P* > 0.05) (Fig. 1D).

### Diagnostic performance of serum HULC and CCAT2

**Figure 2:**
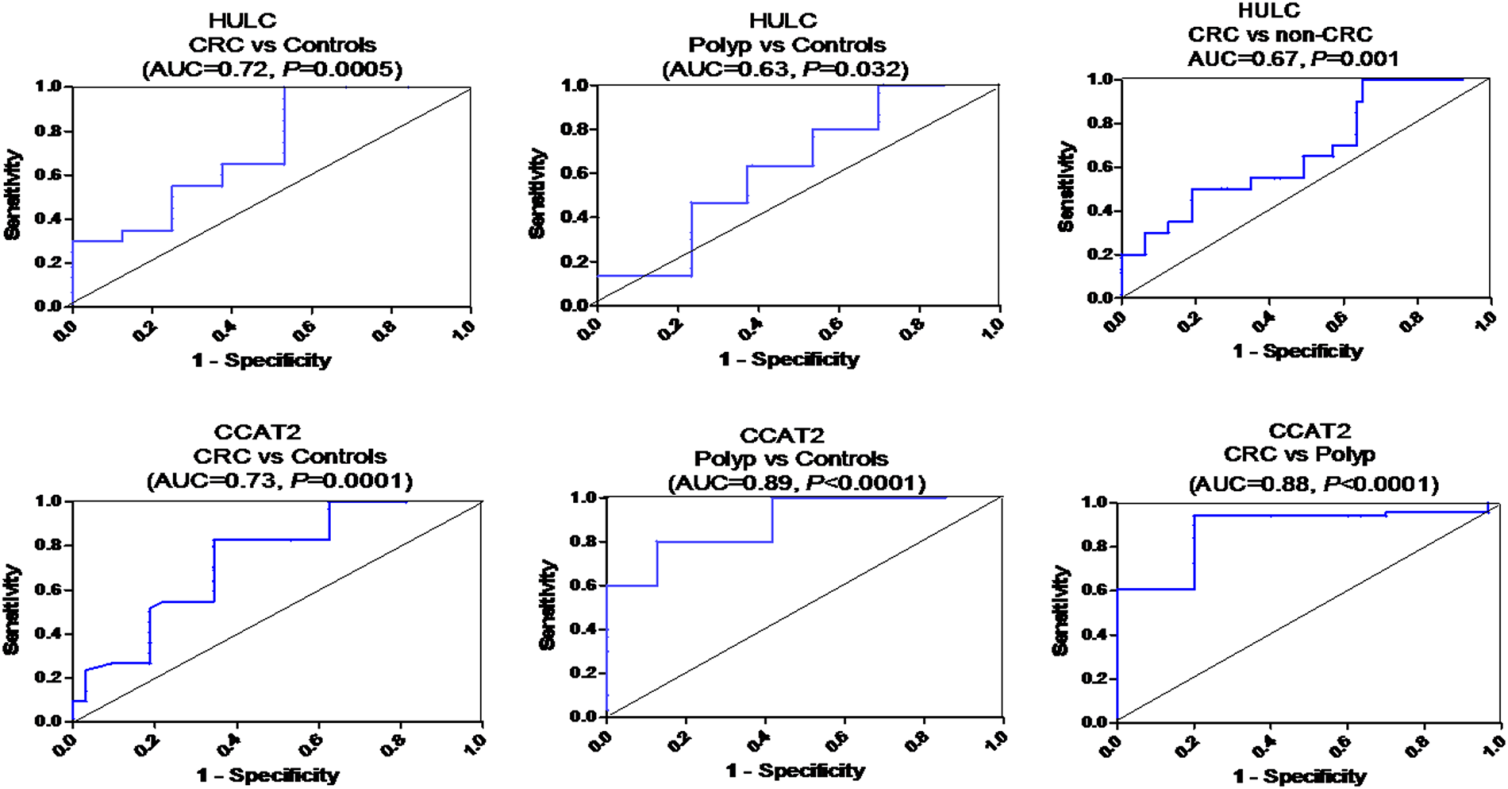
Diagnostic performance of serum HULC and CCAT2. ROC curve analysis of serum HULC and CCAT2 to discriminate studied groups, CRC (n = 120), adenomatous polyps (n = 30), healthy controls (n = 96), and non-CRC groups (n = 126).

ROC analysis revealed that serum HULC discriminated CRC from healthy controls with AUC = 0.72, 95% CI = 0.607–0.834, *P* = 0.0005, with sensitivity = 55%, specificity = 75%, positive predictive value (PPV, certainty to prove CRC) = 73% and negative predictive value (NPV, certainty to exclude CRC) = 57% at a cutoff >4.3 fold. Serum HULC also distinguished AP patients from controls with AUC = 0.63, 95% CI = 0.525–0.738, *P* = 0.032, with sensitivity = 46.7%, specificity = 77%, PPV = 38.8%, and NPV = 82.2% at a cutoff >4 fold. In addition, serum HULC discriminated CRC from non-CRC with AUC = 0.67, 95% CI = 0.576–0.766, *P* = 0.001, with sensitivity = 50%, specificity = 81.3%, PPV = 71.4%, and NPV = 62.9% at a cutoff >4 fold (Fig. 2).

ROC analysis also revealed that serum CCAT2 discriminated CRC from healthy controls with AUC = 0.73, 95% CI = 0.685–0.818, *P* = 0.0001, with sensitivity = 82.5%, specificity = 66.66%, PPV = 75.57%, and NPV = 75.29% at a cutoff >1.5 fold. Serum CCAT2 also distinguished AP patients from controls with AUC = 0.89, 95% CI = 0.827 to 0.956, *P* < 0.0001, with sensitivity = 80%, specificity = 87.5%, PPV = 66.66%, and NPV = 93.3% at a cutoff >8.8 fold. It also distinguished CRC from AP with AUC = 0.88, 95% CI = 0.819 to 0.944, *P* < 0.0001, with sensitivity = 94.7%, specificity = 79.16%, PPV = 95%, and NPV = 80% at a cutoff <11 fold (Fig. 2).

### Results of logistic regression analysis

We conducted univariate and multivariate logistic regression analyses to select the predictor variables associated with CRC risk among non-CRC groups (Table 5). Serum HULC, rs6983267 and rs7763881 genotypes were selected as significant predictor variables in the univariate analysis, with adjustment for age and sex. In multivariate analysis, only serum HULC and rs6983267 turned out to be significant independent predictors of CRC susceptibility.

**Table 5:** Logistic regression analysis to predict the risk of CRC in non-CRC groups.

Parameter	Coefficient	SE	*P* ^a^ value	OR^a^	OR (95% CI)
**Univariate analysis**					
HULC expression	0.129	0.031	<**0.0001**	1.137	1.07–1.207
rs6983267 (GG vs GT/TT)	0.837	0.295	**0.005**	2.3	1.295–3.533
rs7763881 (AC vs AA)	−0.774	0.337	**0.02**	0.46	0.238–0.892
**Multivariate analysis**					
HULC expression	0.136	0.034	<**0.0001**	1.146	1.073–1.224
rs6983267 (GG vs GT/TT)	0.932	0.319	**0.004**	2.538	1.358–4.761
rs7763881 (AC vs AA)	−0.529	0.369	0.152	0.589	0.285–1.215
Constant	−0.657				

Log likelihood of the stepwise multivariate logistic regression model = −144.39, −2 Log likelihood = 288.78, *P* < 0.0001. *P* ^a^ adjusted for age and sex. CRC, n = 120, non-CRC (healthy controls + AP), n = 126. *P* values in bold are statistically significant *P* < 0.05.

### Correlation of rs7763881 and rs6983267 genotypes, serum HULC and CCAT2 levels with clinicopathological data

We examined the prognostic role of studied SNPs and serum lncRNAs in CRC patients (Table 6). No significant correlations were found for rs7763881, rs6983267 genotypes, serum HULC and CCAT2 with tumor-related data, nodal and distant metastases, except a negative correlation between rs7763881 AC genotype and the presence of mucinous adenocarcinoma (adjusted OR = 0.152, 95% CI = 0.035–0.666, *P* = 0.016) after adjustment for age and sex. Serum CCAT2 was positively correlated with serum HULC (r = 0.67, *P* = 0.0007).

**Table 6:** Correlation of rs7763881 and rs6983267 genotypes, serum HULC and CCAT2 expression levels with clinicopathological data of CRC patients.

Parameter	HULC rs7763881			rs6983267			Serum HULC			Serum CCAT2		
	AA	AC	*P* ^a^	GG	GG/TT	*P* ^a^	High	Low	*P* ^a^	High	Low	*P* ^a^
	(n = 32)	(n = 88)		(n = 46)	(n = 74)		expression	expression		expression	expression	
Anatomical site, n (%)			0.79			0.44			1			0.14
Colon	24 (27)	64 (73)		32 (36)	56 (64)		44 (50)	44 (50)		40 (45)	48 (55)	
Rectum	8 (25)	24 (75)		14 (44)	18 (56)		16 (50)	16 (50)		20 (62.5)	12 (37.5)	
Type of tumor, n (%)			0.46			0.68			0.6			1
Adenocarcinoma	32 (27.5)	84 (72.5)		44 (38)	72 (62)		59 (51)	57 (49)		58 (50)	58 (50)	
Others	0 (0)	4 (100)		2 (50)	2 (50)		1 (25)	3 (75)		2 (50)	2 (50)	
Tumor grade, n (%)			0.38			0.176			0.36			0.49
Well/moderate	28 (31)	62 (69)		30 (33)	60 (67)		48 (53)	42 (47)		47 (52)	43 (48)	
Poorly-differentiated	4 (15)	22 (85)		14 (54)	12 (46)		11 (42)	15 (58)		11 (42)	15 (58)	
Mucinous tumors, n (%)			**0.016**			0.165			1			0.72
Yes	6 (60)	4 (40)		6 (60)	4 (40)		5 (50)	5 (50)		6 (60)	4 (40)	
No	26 (24.5)	80 (75.5)		38 (36)	68 (64)		54 (51)	52 (49)		52 (49)	54 (51)	
Nodal involvement, n (%)			0.13			0.3			0.79			0.8
Yes	8 (18)	36 (82)		14 (32)	30 (68)		22 (52)	20 (48)		22 (52)	20 (48)	
No	24 (32)	52 (68)		32 (42)	44 (58)		38 (49)	40 (51)		38 (49)	40 (51)	
Distant metastasis, n (%)			0.09			0.3			0.2			0.8
Yes	2 (10)	18 (90)		10 (50)	10 (50)		13 (65)	7 (35)		9 (45)	11 (55)	
No	30 (30)	70 (70)		36 (36)	64 (64)		47 (47)	53 (53)		51 (51)	49 (49)	

Values are expressed as number (percentage). A cutoff of 3.5 fold for serum HULC and 2 fold for serum CCAT2 typically divided HULC and CCAT2 expression data equally to high or low expression. *P* ^a^ adjusted for age and sex in a logistic regression model. *P* values in bold are statistically significant (*P* < 0.05).

## Discussion

The present study revealed association of rs6983267 at 8q24 and HULC rs7763881 SNPs with the susceptibility for CRC, but not adenomatous polyps. These SNPs were functionally correlated with serum CCAT2 and HULC expression, respectively. In addition, combined presence of these two polymorphisms in our studied population significantly altered the CRC susceptibility, confirming that CRC is predisposed by combination of low-penetrance susceptibility alleles. These results may add to the complex heterogeneity and pathology of CRC and implicate these SNPs, through functional modulation of lncRNAs expression, as potential genetic susceptibility markers for sporadic CRC.

To the best of our knowledge, we provided the first evidence of the association between HULC rs7763881 polymorphism with decreased CRC susceptibility. rs7763881 AC genotype was a decreased risk factor for CRC compared with the AA genotype. This protective role was evident among male patients and younger patients in our study. Similarly, HULC rs7763881 AC/CC genotypes conferred a lower risk for HBV-related hepatocellular carcinoma^17^, and the AC genotype was a protective factor against esophageal squamous cell carcinoma in Chinese population compared with the AA genotype^18^. However, the exact mechanism of rs7763881 and its role in regulating HULC expression was not known.

In this study, we are the first to investigate the functional role of the HULC rs7763881. We found that the AC genotype was associated with lower HULC levels than the AA genotype. This could explain the protective role of rs7763881 by reducing the oncogenic HULC level. These reduced levels could explain the observed negative association between the AC genotype with mucinous adenocarcinoma, a histologic variant characterized by huge amounts of extracellular mucus and is associated with advanced CRC^19^. Notably, HULC expression is regulated by several mechanisms, including promoter methylation, transcription factors, RNA destabilization, lncRNA-lncRNA interaction, and post-transcriptional regulation by miR-203^20^. Additionally, our results propose rs7763881 SNP as a new mechanism regulating HULC expression in cancer.

For rs6983267, homozygocity for the G allele contributed to increased CRC risk. Conversely, heterozygocity (GT) was protective, while the TT genotype wasn’t a significant CRC risk candidate compared with the GG genotype in our study. The GG-associated risk was evident among male patients and older patients (>50 years) in a dominant model. In addition, the GG genotype independently predicted increased CRC risk among non-CRC groups by 2.5 fold in multivariate analysis. Similarly, rs6983267 GG was associated with increased sporadic CRC susceptibility in an Iranian population and individuals with GT genotype had lower risk for CRC^21^. The homozygous G allele of rs6983267 was also associated with a high CRC risk, while the homozygous T allele was a non-risk allele^22^. Furthermore, several studies showed that the rs6983267 GG is related to increased risk of various cancers in several populations^7,23–25^. These findings suggest that the rs6983267 GG genotype is a multicancer susceptibility marker. Conversely, the rs6983267 GT was associated with an increased gastric cancer risk in Chinese population compared with the GG genotype^26^. This discrepancy may be due to differences in the population studied and the tissue type.

Although the rs6983267 has been established as a cancer-related SNP, its biological function remains unclear. An interaction of the risk GG allele with increased MYC expression has been described^10,13^. The GG genotype is highly homologous to the binding site of the transcription factor *TCF4/LEF1*, which enhances MYC transcription^13^, whereas the TT genotype cannot bind the TCF4/LEF protein^27^. However, other studies didn’t clearly find this association^14,28^. Another correlation of rs6983267 with CCAT2 expression in CRC tissues was found^10,11^. However, one study unexpectedly demonstrated higher CCAT2 level with the non-risk TT genotype^11^. In our study, we further assessed the correlation of rs6983267 with CCAT2 expression. We found that rs6983267 GG was associated with higher serum CCAT2 than GT, TT or GT/TT variants, although a statistical significance wasn’t reached for the TT. Our results are consistent with previously reported^10^ and confirm that the SNP-conferred CRC risk may be through potential regulation of CCAT2.

Regarding the prognostic role of rs6983267, we found no association of this SNP with tumor-related characteristics, nodal and distant metastases. Our results are consistent with previous reports in CRC and prostate cancer^11,29,30^, while contrasting others that showed a correlation of rs6983267 with node metastasis in endometrial cancer and distant metastasis in inflammatory breast cancer^24,30^. It seems probable that the relation of the cancer-associated rs6983267 with tumor aggressiveness may depend on the tissue type.

Aberrantly expressed lncRNAs in tumor tissues have emerged as promising biomarkers for cancer diagnosis and prognosis^5^, but the invasive nature of biopsies may limit their use. Few studies have addressed circulating lncRNAs as non-invasive markers for CRC^31,32^. Herein, we demonstrated that serum CCAT2 and HULC were differentially expressed between CRC patients and controls and/or adenoma patients, and discriminated CRC from other groups with moderate sensitivity and specificity, suggesting serum HULC and CCAT2 as novel potential early biomarkers for CRC diagnosis. However, serum HULC, but not CCAT2, was significantly upregulated in CRC vs non-CRC groups and distinguished the two groups by ROC analysis. Interestingly, serum HULC independently predicted the risk of CRC diagnosis among non-CRC groups in multivariate analysis. These results implicate serum HULC as reliable non-invasive early biomarker and possible therapeutic target for CRC treatment. Perhaps combining HULC with other tumor markers may improve the early diagnosis of CRC, however‚ this needs further investigation.

The observed upregulation of serum CCAT2 and HULC in CRC is consistent with their oncogenic roles^10,16^. This upregulation agreed with previous reports in CRC tumor tissues and cell lines^10,16^, which could be reflected in the serum. Indeed, lncRNAs are packaged into secreted microparticles, specifically exosomes^33^. We also found a significant positive correlation between serum CCAT2 and HULC, suggesting their concomitant expression in CRC. However, we found no correlations between these lncRNAs and tumor-related data, nodal and distant metastases. While several studies reported that CCAT2 and HULC were associated with nodal and/or distant metastases in CRC and several cancers^10,16,34–37^, others found no association^11,38,39^. This controversy could be due to differences in the samples used: tissue or serum, the type of lesion analyzed: primary or metastatic, patients’ tumor stages, and number of patients with metastasis. In our study, serum samples were collected from patients during CRC screening, where most tumors were limited, neither nodal (63.3%) nor metastatic (83.3%).

As sporadic CRC mostly develops in AP through the adenoma-carcinoma sequence^40^, we tested if the studied SNPs or lncRNAs contribute to CRC through the development of adenoma. We failed to find an association between neither rs6983267 nor rs7763881 with the susceptibility to AP. Conversely, rs6983267 was significantly associated with colorectal adenoma in a large case-control study of 1,477 individuals with colorectal adenoma and 2,136 controls^8^. However, we couldn’t verify this association in the studied Egyptian population perhaps due to the comparably small sample size of our study. Intriguingly, serum HULC and CCAT2 were upregulated in AP patients compared with controls and were differentially expressed between CRC and AP groups, however, a statistical significance wasn’t reached for HULC. These results implicate HULC and CCAT2 dysregulation as key pro-tumorigenic factors in CRC initiation in colorectal adenoma. AP is a precancerous condition, and its progression to cancer development requires additional molecular modifications, particularly increased cell proliferation^41^. Indeed, HULC and CCAT2 promote different pro-tumorigenic phenotypes, such as cell survival, proliferation and invasion *in vitro* and *in vivo* in many cancers^16,20,42,43^. Specifically, the paradoxically higher CCAT2 levels in AP than CRC in our study probably suggest that AP may need high level of carcinogenic factors like CCAT2 to convert to carcinoma.

Colonoscopy is the gold standard for CRC screening, but it is an invasive method. Our study proposed new blood-based non-invasive genomic markers based on PCR techniques which appear both reproducible and cost-effective. However, our study is limited by the relatively small sample size, and being a hospital-based case-control study, selection bias might have ineluctably occurred. Extended studies with independent larger samples are required to validate our results, and large-scale investigations across different populations should be undertaken. Finally, the interaction of the studied genes with environmental risk factors of CRC should be evaluated; nevertheless our results implicate rs6983267 at 8q24 and HULC rs7763881 as potential genetic markers of CRC susceptibility that correlate with CCAT2 and HULC expression, respectively. Serum CCAT2 and HULC could serve as novel potential early diagnostic biomarkers for CRC, with rs6983267 and serum HULC could predict the risk of CRC diagnosis among non-CRC groups. Our data have potential implications for CRC screening, genetic counseling, and hold promise for large-scale application.

## Materials and Methods

### Patients

This case-control study included 150 adult (>18 years old) Egyptian patients who attended the Gastrointestinal Endoscopy Unit in Kasr AL-Ainy Hospital, Cairo University and referred to colonoscopic examination for lower GIT symptoms‚ including chronic diarrhea, chronic constipation, alternating bowel habits and bleeding per rectum; alarming symptoms and signs for CRC, including significant unexplained weight loss and unexplained anemia; screening for CRC; and metastases proved to be adenocarcinoma and were suspected to have CRC.

Patients were divided into 120 CRC cases and 30 cases with AP based on positive colonoscopy and the diagnosis was confirmed by pathology results. All patients were subjected to full history taking and clinical examination, including routine laboratory investigations: complete blood count, erythrocyte sedimentation rate (ESR), carcinoembryonic antigen (CEA) assay, stool analysis, fecal occult blood test, and liver biochemical profile; full colonoscopy; and imaging using abdominal ultrasound and computed tomography to stage CRC according to American Joint Committee on Cancer (AJCC, 2010)^44^. Patients previously received chemo- and/or radiotherapy for CRC, diagnosed with inflammatory bowel disease (IBD), had cancer at any other site at the time of selection or a history of recurrent tumors were excluded.

A total of 96 apparently healthy controls were age- and sex-matched to the patient population. Controls had negative colonoscopy results for malignancy, polyps or IBD and had no personal or family history of familial adenomatous polyposis and hereditary non-polyposis CRC.

Written informed consent was obtained from all patients and controls. The study protocol and informed consent were approved by the ethics committee of the Faculty of Pharmacy, Cairo University (BC2058) and conformed to the ethical guidelines of Helsinki Declaration.

### DNA extraction and genotyping

Genomic DNA was extracted from whole EDTA blood samples from all subjects using the QIAamp DNA MiniKit (Qiagen, Valenica, CA) according to the manufacturer’s instructions. The yield was measured by NanoDrop2000 (Thermo scientific, USA). Genotyping was performed using real-time PCR with the TaqMan allelic discrimination assay using predesigned primer/probe sets for rs6983267 (G/T) [C_29086771_20] and rs7763881 (A/C) **[**C_29335152_10](Applied Biosystems, USA). DNA amplification was performed in a 25 μl volume containing 12.5 μl TaqMan master mix, 1.25 μl primer/probe, 1 μl DNA, and 10.25 μl H_2_O. Real-time PCR was performed using a Rotor gene Q System (Qiagen) with the following conditions: 95 °C for 10 min, followed by 45 cycles at 92 °C for 15 s and 60 °C for 90 s. Fluorescence was measured at the end of every cycle and at the endpoint.

### Serum lncRNAs assay

Total RNA was extracted from serum by miRNeasy extraction kit (Qiagen, Valenica, CA) using QIAzol lysis reagent according to the manufacturer’s instructions. RNA quality was determined using NanoDrop2000 (Thermo scientific, USA). Reverse transcription (RT) was carried out on 60 ng of total RNA in a final volume 20 µl RT reactions using RT^2^ first strand Kit (Qiagen, Valenica, CA) according to the manufacturer’s instructions. The RT products were diluted with 50 µl RNAase-free water before real-time PCR. Serum expression levels of the studied lncRNAs were evaluated using GAPDH as internal control using customized primers and Maxima SYBR Green PCR kit (Thermo, USA) according to the manufacturer’s protocol. The primer sequences were as follows: CCAT2-forward 5′-CCCTGGTCAAATTGCTTAACCT-3′, CCAT2-reverse 5′-TTATTCGTCCCTCTGTTTTATGGAT-3′, and HULC-forward 5′-TCATGATGGAATTGGAGCCTT-3′, HULC-reverse 5′-CTCTTCCTGGCTTGCAGATTG-3′, and GAPDH-forward 5′-CCCTTCATTGACCTCAACTA-3′, GAPDH-reverse, 5′-TGGAAGATGGTGATGGGATT-3′. Briefly, real-time PCR was done on 20 µl reaction mixture prepared by mixing 10 µl master mix, 1 µl forward primer, 1 µl reverse primer, 2.5 µl diluted cDNA, and 5.5 µl RNAase-free water using Rotor gene Q System (Qiagen) with the following conditions: 95 °C for 10 min, followed by 45 cycles at 95 °C for 15 s and 60 °C for 60 s. The cycle threshold (Ct) is the number of cycles required for the fluorescent signal to cross the threshold in real-time PCR. Gene expression relative to internal control (2^−∆Ct^) was calculated. Fold change was calculated using 2^−∆∆Ct^ for relative quantification^45^.

### Statistical analysis

Statistical analyses were performed using computer program Statistical Package for the Social Science (SPSS, Chicago, IL) software version-15 for Microsoft Windows and GraphPad Prism-5.0 (GraphPad Software, CA). Values were expressed as mean ± standard deviation (SD), mean  (95% confidence interval, CI) or number (percentage) when appropriate. Categorical data were compared by Chi square or Fisher’s exact test when appropriate. Continuous variables were compared using student’s t-test or one way ANOVA followed by Tukey’s post-hoc test when appropriate. The diagnostic accuracy of lncRNAs was evaluated by receiver-operating-characteristic (ROC) analysis and the area under the curve (AUC) was calculated. AUC <0.6 was considered non-significant, between 0.7–0.89 was considered potential discriminator, whereas AUC >0.9 was considered significant discriminator. Univariate and multivariate logistic regression analyses were done to identify predictor variables associated with the risk of CRC. For adjustment of the data to the confounding factors, age and sex were included as covariates. Significant predictor variables in the univariate analysis were included in a stepwise forward multivariate analysis (*P* < 0.05 for entering the model and *P* < 0.1 for removal from the model) to determine the final predictor variables for the probability of being diagnosed with CRC. An internal 10-fold cross-validation was conducted to confirm the reproducibility of the results. Correlations were determined by Pearson correlation. *P* < 0.05 was considered significant, with a 95% CI.

### Data availability

All data generated or analyzed during this study are included in this article and its supplementary information files.

## Electronic supplementary material

Supplementary Information
