# Supplementary material for: Association of rs6983267 at 8q24, HULC rs7763881 polymorphisms and serum lncRNAs CCAT2 and HULC with colorectal cancer in Egyptian patients
Source: Sci Rep. 2017 Nov 24;7:16246. doi: 10.1038/s41598-017-16500-4 (PMC5701156; doi:10.1038/s41598-017-16500-4)
Supplement: Supplementary file 1 — Supplementary Information [file 41598_2017_16500_MOESM1_ESM.doc]

**Association of rs6983267 at 8q24, HULC rs7763881 polymorphisms and serum lncRNAs CCAT2 and HULC with colorectal cancer in Egyptian patients**

**Olfat G. Shaker^1^, Mahmoud A. Senousy^2^, Eman M. Elbaz^2^**

^1^Medical Biochemistry and Molecular Biology Department, Faculty of Medicine, Cairo University, Cairo, Egypt

^2^Biochemistry Department, Faculty of Pharmacy, Cairo University, Cairo, Egypt

**Supplementary Information**

**Table S1 Minor allele frequencies of the studied SNPs**

|  | MAF in our studied population | Global MAF^$^ | Highest population MAF^$^ |
| --- | --- | --- | --- |
| rs6983267 | T=0.43 | T=0.39 | T=0.5 |
| rs7763881 | C=0.44 | C=0.42 | C=0.49 |

MAF, minor allele frequency. ^$^according to Ensembl GRCh37 release 89 - May 2017 ©.

**Table S2 Hardey-Winberg equilibrium of rs6983267 in CRC group**

|  | Observed | Expected | *P* |
| --- | --- | --- | --- |
| GG | 38.3 | 38.4 | 0.86 |
| GT | 46.7 | 47.2 |  |
| TT | 15 | 14.4 |  |
